# The Diagnosis and Treatment of Appendicitis*Read before the Kentucky Midland Medical Society, at Shelbyville, Ky., January 13, 1898.

**Published:** 1898-03

**Authors:** Louis Frank

**Affiliations:** Visiting Gynecologist to the Louisville City Hospital, Louisville, Kentucky


ATLANTA

### Medical and Surgical Journal.

VOL. XV. MARCH, 1898. No. 1.

L. B. GRANDY, M.D., DUNBAR ROY, M.D., EDITORS.

M. B. HUTCHINS, M.D., BUSINESS MANAGER.

### ORIGINAL COMMUNICATIONS.

### THE DIAGNOSIS AND TREATMENT OF APPENDICITIS.*

BY LOUIS FRANK, M.D.,

Visiting Gynecologist to the Louisville City Hospital, Louisville, Ky.

The papers which have been written upon appendicitis have been almost invariably read before surgical societies and discussed by surgeons. Rarely do we hear the ideas of the general practitioner upon this subject. I anticipate censure or at least disagreement upon the part of many of the members in my consideration of the treatment of this dangerous trouble; but it is the experience of the doctor that we are after. Surgeons want to know how many cases he has seen which have gone through an attack without operation; we want to know how many cases he has seen upon which no operation has been performed that have had a recurrence; we want to know what has been the ultimate outcome in these cases; how many of them were really or permanently cured. I feel sure that shall meet with opposition when I speak of operating upon all cases of appendicitis. I might say here that I believe that as soon

* Read before the Kentucky Midland Medical Society, at Shelbyville, Ky., January 13, 1898.

as the diagnosis of appendicitis is made, the case is no longer a medical case; at the same time I recognize the fact that many patients positively refuse to have the surgeon called in. To this class of cases I will also devote a little attention.

The diagnosis of appendicitis should be a comparatively easy matter, though not always as much so as some would have us believe. There may be great difficulty at times in making the diagnosis, though this is far less frequent in the male than in the female. In the male I think we may say that 99 per cent. of all cases of peritonitis, not due to trauma, are appendiceal in origin; but we should not look upon all pains that occur in the abdomen or that are referred to the abdomen as due to peritonitis. Appendicitis may be confounded with several affections. For instance, I had a case under observation which had been treated for a week for appendicitis, the patient being a man about fifty-six or fifty-eight years of age; I was called to see the patient with the idea of operating. I could not agree in the diagnosis and was inclined to look upon the case as one of renal colic. The patient was put upon treatment and my diagnosis was verified by his passing two days later a small renal calculus. This man had rigidity, pain, vomiting, constipation, with pulse increased in rapidity, due however to pain and not to elevation of temperature. There were other symptoms, however, which in connection with his age made me feel sure his trouble was not appendiceal. Gall-stones have also been mistaken for appendicitis. We should analyze our cases very carefully. The tendency now is to diagnose all acute abdominal troubles as appendicitis. This is due to over-zealousness, and careful consideration of each case will prevent it. I think that few cases of appendicitis go undiagnosed, and those that do have as a rule been improperly treated. This calling every abdominal pain appendicitis has also done much to prejudice people against operation, and also physicians, as they look upon them as cured cases. They are the cases that have no recurrence. The symptoms of appendiceal diseases are so constant that by careful examination differentiation is most always possible. Among the most important of these symptoms are pain referable in almost all cases to the McBurney point, tenderness and rigidity in the right iliac fossa; the other symptoms which we would mention are vomiting, constipation, distention, tumor, rapidity of pulse and elevation of temperature.

Let us analyze the symptoms separately: The first symptom which is usually present is pain. This pain at first may be rather indefinite in location; it may be present about the umbilicus; it may be present in the epigastric region, or it may vary in its location, first here and there; at first the character is that of acute indigestion or intestinal colic. Soon, however, it becomes localized in the right iliac fossa. This pain is due to pressure upon the branches of the sympathetic which are distributed in the swollen appendix. Pain is always present at some time during the course of the disease. It is always present primarily; later in some cases it may entirely disappear, for reasons which will be given directly. Tenderness like pain may always be elicited by pressure over the appendiceal region. Rarely it exists on the left side. This tenderness, in the vast majority of cases, is most intense and can be located exactly at the McBurney point, namely, a point midway between the umbilicus and the anterior superior spinous process of the ilium. Like pain, it may in some cases disappear. It is always present, however, unless there has been a rupture and the patient is in shock; when rupture occurs both pain and tenderness may disappear entirely. These two symptoms, with rigidity of the muscles upon this side, are the most important of all. If we compare the right and the left side in an appendicitic patient, that is, before peritonitis has become diffuse, while it is still localized in the right iliac fossa, whether there is an abscess or not, we find that nature has thrown out her guards in the form of a stiff, rigid muscle. This rigidity is so marked that it is absolutely impossible to do anything in the way of palpating for presence of a tumor.

Now with these important symptoms we have a change in the pulse; we have the pulse of peritonitis; it may not be increaeed much in rapidity, but its character is changed in a marked degree. And just here I would say that it is not the rapidity of the pulse upon which we lay stress, but upon the character of the pulse itself. We find the pulse hard, bounding, throbbing as it were, and one that it is impossible to compress or to make entirely disappear.

We feel it jumping under the fingers as an unruly horse pulling against the bridle. The pulse rate may be very little increased, or it may run up very rapidly.

Now as to temperature: The temperature will depend upon the character of the infection. I do not lay any stress upon the temperature in connection with appendicitic cases when considered by itself. Taken in conjunction with other symptoms it is sometimes a valuable means of diagnosis. I have seen a most severe case of appendicitis where the temperature range was not higher than 100° F.; I have seen cases where it did not reach 100° F. until the time of rupture. I have seen others where there was not nearly so much disease about the appendix, where the temperature was as high as 103° and 104° F. Morris has said that with the colon bacillus infection we need not look for high temperature; with pyogenic organisms we may look for it.

Vomiting is usually an early symptom, and is often at first, though not always persistent; first the contents of the stomach, afterwards bile. As the case becomes more and more septic this vomited matter contains little black particles, not the coffee-ground vomit, however; but it is streaked with black and contains little black specks. The vomiting may then be either biliary in character, or it may consist entirely of mucus. This black streaking is due to hemorrhages (petechial) from the mucous membrane of the stomach. With increase in peritonitis the vomiting may be stercoraceous.

*Constipation:* Constipation is present in most of the cases of appendicitis, and usually persists during the initial stage of the attack, or at least until the administration of some saline. However, we may find diarrhea from the incipiency of the disease, or as a symptom of sepsis. Constipation may be very obstinate at times, refusing to yield to any treatment; there is intestinal paralysis due to toxemia. This may lead to mistake in diagnosis as in a case I now call to mind, which was treated primarily for obstipation, then for obstruction, in which an operation (although it was fatal) proved the case to be one of the worst sort of appendicitis.

*Distention:* Distention begins from the very start. It may be very slight, or it may be marked, usually progressing with the continuation of the disease. Upon inspection the distention may not be marked, but if we percuss over the abdomen we will find there is a diffuse tympanitic note which extends up under the ribs, and we find it on the right side where we should find liver dulness. By percussing upon either side over the floating ribs we can make out this distention, when it is at times not apparent by inspection nor so marked by percussion over the lower abdomen.

These are the most important symptoms, and the only ones, I take it, that it is necessary to dwell upon, and their importance I should say was in the order named.

There is one symptom to which a great deal of prominence has been attached, far more than it deserves, and upon its presence or absence the diagnosis is ofttimes, though it should not be, dependent. I refer to tumor. On account of the rigidity a tumor can not always be made out even when present. We may get over the area of the effusion, or of the adhesions, or of the abscess sac, whichever may be present, upon percussion, a dull note. In many cases there is no tumor at all present. These I think are the most dangerous of all the cases, and are those where there have not been many adhesions and where, when the appendix ruptures, rupture is into the free cavity. Those cases where there is a tumor, particularly if of any size, are really far less dangerous than the other class; therefore, I think presence or absence of tumor of little diagnostic value. With rupture where there are adhesions shutting off the appendix from the general cavity, or with formation of an abscess, a tumor, when one is present, may rapidly increase in size and may reach apparently enormous proportions. These are the cases that are operated upon merely by opening the abscess, washing out and draining. They too frequently go on to recurrences notwithstanding the evacuation of pus, because the appendix, the seat of the original trouble, is still present.

A careful consideration of these symptoms will render a diagnosis easy in the male, but we should never jump to a conclusion; we should make our diagnosis carefully always by exclusion.

In the female, while appendicitis is not nearly so frequent as in the male, this being due to the increased blood supply to the appendix through Cleido's ligament, the diagnosis may at times present more difficulties. We have here to differentiate tubal disease, and some cases which I have recently seen have impressed the importance of a careful examination in arriving at an opinion Of course there are cases where there is no difficulty, but we should always examine carefully into the menstrual history of the patient, be she maid, wife or widow. If there is the least doubt we should examine before operation under anesthesia and exclude absolutely the possibility of disease of the uterine appendages. We should also bear in mind that disease of the appendix and of the uterine appendages may coexist in the same patient, the trouble in the generative organs being due to secondary infection from the appendix, or we may have them occurring independently of each other. I do not believe it is possible for disease of the appendix, except possibly adhesions due to extensive inflammatory trouble, to result from primary disease of the tubes or ovaries. By that I mean to say that we do not have appendicitis occurring secondarily to troubles about the uterine appendages. Those cases where the most difficulty arises are cases where there is an extremely long vermiform appendix, one which hangs down over the brim of the pelvis, thus referring the pain lower down than usual, and showing by bimanual examination the presence of a mass high up in the pelvis upon the right side. The mass is, however, not connected with the uterus, and that organ is movable. Careful examination will always enable us to differentiate from tubal disease.

*As to treatment:* I have indicated previously, that this disease, as soon as diagnosis has been made, is a surgical one. "We might divide the treatment into medical and surgical." I do not mean by this that I think the treatment of a case of appendicitis should ever be medical in character, but I recognize the fact that oftentimes patients are averse to having an operation performed ; some will positively refuse, others desire to delay it as long as possible.

The very word "operation" will frighten some half to death ; they dread the knife, and I have seen people who would prefer death to the surgeon. Why is this? It should not be, for the surgeon is really in these cases a life-saver. It is prejudice, due to lack of education or ignorance, and at times, I regret to say, to the physician, who fears either to lose his fee, his influence and prestige by calling the surgeon, or by not being able to perform the work himself; or he may fear death from the operation and censure on account thereof from the community. Should this be? No! Let us educate the people to the true value of the conservative surgeon. In other instances it is impossible to obtain a surgeon at once, and for these cases there must be some treatment. The practitioner is expected to do something, and he must do something. There is one thing, however, he should not do—and upon this too much stress cannot be laid—that is to administer opium. There is never a time in the treatment of appendicitis when opium is indicated. I believe that lives would have been saved had this been borne in mind in treating such patients. I recognize full well that it is at times difficult to resist the temptation to administer a few doses of opium, especially after you have made the diagnosis, after you have convinced yourself, and there is no longer any doubt as to the nature of the trouble. You say to yourself, here is a case of appendicitis, why not give the patient a little relief by a hypodermic; it acts so nicely and is so quickly done? The results are so perfect that it is at times almost irresistible. It should not, however, be yielded to. We should resist the temptation, not because it obscures our diagnosis, it being already made, but because pain being allayed we are at once given a false sense of security. We may then think that our patient may get along without an operation. We go on from day to day giving one or two hypodermics, being all the time convinced that we have a case of appendicitis, but that the symptoms have subsided, and our patient goes on to death simply because we could not refuse their pleadings or had not the courage of our convictions.

To my mind medical treatment should consist only in purgation with salines, or an attempt at purgation, the administration of enemata to empty the bowels, and hot applications; nothing more unless at times strychnia as a stimulant. These things may relieve the pain. If there is any curative value, or any benefit to be derived from medical treatment, it will be derived from purgation, getting rid of noxious products, keeping the bowels open, and if there are any cases that can be cured by medical or medicinal means, it will be these. I have seen pain subside very quickly after free purgation; I have seen the symptoms almost disappear, and have seen cases which got well, or at least which recovered from the attack at the time, by the administration of salts. True the attack sooner or later recurs. The fact that I have seen cases pass through an attack without operation weighs not one bit in the balance with me as compared with the good to be derived from operative interference.

The question when to operate has been a great deal discussed ever since appendicitis was recognized as a surgical trouble. I would say with Morris, operate as soon as the diagnosis is made. "Isolate an infected appendix, from the peritoneal cavity, just as you would isolate a case of diphtheria in a healthy community." One is as essential as the other. There is only one answer to the question in my mind when to operate, and that is at once. The presence of an appendix previously inflamed is a constant menace to health and to life. There is only one class of cases upon which I would not operate, and of late I am even at times inclined to operate upon these, and that is where the case is desperate, where there is rupture, where the patient is on the brink of the grave, as it were, where you feel sure that notwithstanding the operation recovery is almost impossible. These are the cases, however, where we are urged to operate, and they are the ones which have brought appendiceal surgery into disrepute with the laity. They are the ones where we see reported in the newspapers, "Death from Appendicitis." I may say in passing, that these newspaper reports have cost many lives by giving rise to prejudice on the part of the laity; they think that all cases of appendicitis operated upon die. They never hear of the ones that are cured, and if we refuse to operate upon this class they would hear even less of those that die.

As to the operation itself: I take it that you are all familiar with the technique, or sufficiently so as not to necessitate a description of the operation by me. But there is one thing that I must dwell upon, and that is the question of adhesions and their treatment, and the disposition of the appendix. The appendix should be removed in every case at the primary operation, unless the patient is in such a bad condition that it would not do to prolong etherization to search for and remove it. Again, in the practice of the country doctor, where an operation is done without sufficient assistance, and without sufficient preparation for a thorough operation, leaving the appendix may at times be excusable. An appendix left behind is a source of future trouble, and for that reason I think it is safer to break up the adhesions, search for the offending organ and take it away. It should be taken away as close to the bowel as is possible. I say as is possible, because there may be cases where we cannot get the colon sufficiently up, or where for other reasons we are not able to treat the appendix in this manner. A portion of an infected appendix left behind may form a focus for abscesses which may discharge later, either into the bowel or externally, or into the peritoneal cavity. I have heard expressions such as this in speaking of appendiceal operations: "I did not remove the appendix because it had sloughed off." Even when the appendix has sloughed off, it should be searched for, the adhesions in which it lies embedded broken up, and it should be removed. We do not know that it has sloughed off, however, unless we trace the colon down to the appendiceal attachment and note its presence or absence. This requires separation of adhesions. Even after the appendix is removed I think it well to separate all adhesions, and this is especially true if there has been any pus formation. All pus pockets must be opened, washed out and possibly drained. The reasons for this are as evident as those for the removal of the appendix in every case.

As to the necessities for drainage: Each case must be a rule unto itself, and the after treatment will depend upon the character of the operation and the condition found. The general indications are about the same as in any other abdominal section.

*129 West Chestnut street.*

---

Teacher—Now, children, we will have our verses. First Small Child (repeating verse)—He that hath ears to hear let him hear. "Very good. Now the next little boy." Small Boy (taking his cue)—He that hath a noth to shmell let him shmell."—*Ex.*

